# Electrophysiological sensors reveal silicon–selenium interaction of dynamic leaf intracellular water–nutrient metabolism in rice under cadmium stress

**DOI:** 10.1080/15592324.2025.2547384

**Published:** 2025-08-23

**Authors:** Xiangyong Gu, Antong Xia, Yanyou Wu, Twagirayezu Gratien, Jing Fan, Chen Wang, Dongshan Xiang, Kun Zhai, Xinjian Song

**Affiliations:** a Hubei Key Laboratory of Selenium Resource Research and Biological Application, Hubei Minzu University, Enshi, China; b School of Chemical and Environmental Engineering, Hubei Minzu University, Enshi, China; c State Key Laboratory of Environmental Geochemistry, Institute of Geochemistry, Chinese Academy of Sciences, Guiyang, Guizhou, China

**Keywords:** Silicon–selenium interactions, cadmium stress, plant electrophysiological sensors, intracellular water–nutrient metabolism

## Abstract

Silicon (Si^4+^) and selenium selenite (Se^4+^) exhibit antagonistic effects on cadmium (Cd^2+^). While plant electrophysiological sensors can quantify intracellular water and nutrient metabolism, the dynamic interactions between silicon and selenium in rice under cadmium stress remain unclear. This study focused on a rice variety (Yixiangyou 876, Enshi, Hubei Province, China), examining growth, photosynthesis, Se and Cd transport, and intracellular water and nutrient metabolism under varying silicon concentrations. Compared to Cd stress alone, the application of MSi (10 mM Si^4+^ and 8 μM Se^4+^) increased rice growth, significantly increasing water transfer rate (WTR), nutrient active translocation capacity (NAC), and nutrient transfer rate (NTR) by 148.57%, 192.01%, and 148.57%, respectively. This synergistic treatment promoted Se translocation and decreased Cd concentration. In contrast, HSi (15 mM Si^4+^ and 8 μM Se^4+^) suppressed rice growth, decreasing intracellular water holding capacity (IWHC) and NAC by 33.21% and 46.52%, respectively. Thus, 10 mM Si^4+^ in combination with Se^4+^ improved growth, photosynthesis, and intracellular water and nutrient transfer capacity in rice leaves and mitigated cadmium transport. This study provides scientific evidence for Enshi selenium-enriched cadmium-reduced rice. Its core value lies in converting complex physiological processes into quantifiable electrical signals, enabling researchers and farmers to precisely regulate the absorption and metabolism of selenium and cadmium in rice, ultimately achieving the agricultural goal of "high-quality, safe, and efficient" selenium-enriched agriculture.

## Introduction

Cadmium (Cd) is a toxic heavy metal that poses significant risks to agricultural productivity, particularly in rice, a staple food for over half of the world's population.^1^ Cd stress can result in decreased growth, photosynthesis, and nutrient imbalances in rice plants.^2^ Consequently, preventing Cd stress in rice is crucial for food security and environmental safety.^3^^,^^4^​​​​​​ Enshi (29°07′10″–31°24′13″N, 108°23′12″–110°38′08″E, Hubei Province, China), known as the "selenium (Se) capital of the world," contains soil enriched with 4.43% Se.^5^ However, the high Cd content in Se-rich soils presents a significant challenge to the sustainable development of Se-enriched industries.^6^

Rice is a typical silica-loving plant, and both silicon and Se have been shown to counteract Cd transport in rice.^7^ Specifically, silicon not only inhibits the transport of Cd to the shoots,^8^ but also promotes iron translocation in the roots, thereby reducing the bioavailability of Cd in the soil.^9^^,^^10^ Additionally, Se can compete with Cd for binding sites, reducing Cd translocation in plants.^11^ While the beneficial roles of silicon and Se in Cd antagonism are evident, the interaction between silicon and Se in dynamic intracellular water‒nutrient metabolism under Cd stress in rice remains unclear.

Previous studies have utilized methods such as inductively coupled plasma mass spectrometry (ICP-MS),^12^ hydride generation-atomic fluorescence spectroscopy (HG-AFS),^13^ and hyperspectral imaging (HSI)^14^ to examine Cd^2+^ translocation in plant species. However, the dynamic intracellular water‒nutrient metabolism in plants remains unexplored. Fortunately, plant electrophysiological techniques have emerged as innovative tools for investigating dynamic intracellular water‒nutrient metabolism in crops such as tomatoes,^15^ potatoes,^16^ and oilseed rape.^17^ By employing plant electrical sensors, including intrinsic capacitance (ICP), intrinsic resistance (IR), intrinsic impedance (IZ), intrinsic capacitive reactance (IX_C_), and intrinsic inductive reactance (IX_L_),^18^ it is possible to assess various aspects of intracellular water‒nutrient metabolism, such as intracellular water holding capacity (IWHC), intracellular water use efficiency (IWUE), intracellular water holding time (IWHT), and water transfer rate (WTR),^19^ as well as nutrient translocation rate (NTR), nutrient translocation capacity (NTC), active transport flow of nutrients (UAF), and nutrient active translocation capacity (NAC).^20^^,^^21^ These plant electrophysiological sensors provide a novel approach for understanding silicon‒Se interactions in dynamic intracellular water‒nutrient metabolism in rice under Cd stress.

This study focused on the rice variety Yixiangyou 876, a major rice cultivar from the Se-rich region of Enshi (Hubei Province, China). At varying silicon–Se levels, growth, photosynthesis, total Se and Cd transport, and intracellular water‒nutrient metabolism in rice leaves under Cd stress were investigated using electrophysiological sensors. This study aims to address the following questions: (1) to elucidate the interactions between silicon and Se on rice growth and Cd translocation, and (2) to clarify the intracellular water‒nutrient metabolism at different silicon‒Se levels in rice leaves under Cd stress, based on plant electrophysiological sensors.

## Materials and methods

### Plant materials

In this experiment, rice seedlings (Yixiangyou 876, sourced from Enshi City, Hubei Province, China; rice is in the seedling stage) were selected. The roots of the seedlings were rinsed three times with distilled water before being transplanted into plastic pots (100 cm × 18.5 cm × 8 cm) and cultivated with a modified Hoagland's nutrient solution at 8:00 a.m., ending at 7 p.m., lasting for 21 d.^22^ (The plant is grown indoors with temperature and light control.)

### Silicon–selenium–cadmium treatment

After two weeks, the rice seedlings were transplanted, and Si^4+^, Se^4+^, and Cd^2+^ were added using SiO_2_, CdCl_2_, and Na_2_SeO_3_. Six experimental groups were established (*n* = 3 pots/group, as outlined in Table 1). The Ck group treated rice seedlings without selenium, cadmium, and silicon, rice seedlings were treated with 6 μM Cd^2+^ in the Cd group, rice seedlings were treated with 8 μM Se^4+^ and 6 μM Cd^2+^ in the Sc group, rice seedlings were treated with 3 mM Si^4+^, 8 μM Se^4+^, and 6 μM Cd^2+^ in the LSi group, rice seedlings were treated with 10 mM Si^4+^, 8 μM Se^4+^, and 6 μM Cd^2+^ in the MSi group, rice seedlings were treated with 15 mM Si^4+^, 8 μM Se^4+^, and 6 μM Cd^2+^ in the HSi group and the rice was harvested after 21 d.

**Table 1.** Treatment of Si^4+^, Se^4⁺^, and Cd²⁺.

Group	Treatment (μM, mM)	
Ck	Se^4+^: Cd^2+^ : Si^4+^	0 μM: 0 μM: 0 mM
Cd	Se^4+^: Cd^2+^ : Si^4+^	0 μM: 6 μM: 0 mM
Sc	Se^4+^: Cd^2+^ : Si^4+^	8 μM: 6 μM: 0 mM
LSi	Se^4+^: Cd^2+^ : Si^4+^	8 μM: 6 μM: 3 mM
MSi	Se^4+^: Cd^2+^ : Si^4+^	8 μM: 6 μM: 10 mM
HSi	Se^4+^: Cd^2+^ : Si^4+^	8 μM: 6 μM: 15 mM

### Growth parameters

Use scissors to cut the roots, stems, and leaves of the rice, wash them, and measure the growth parameters of the rice using an analytical balance (0.0001 g, AR124CN, Shanghai, China) (Including root biomass, stem biomass, and leaf biomass).

### Photosynthesis

The second or third fully expanded leaves of the rice plants were selected for photosynthesis analysis between 9:00 and 11:00 a.m., measuring parameters such as net photosynthetic rate (Pn), stomatal conductance (G_S_), intercellular CO_2_ concentration (C_i_), and transpiration rate (Tr) using a Li-6400XT Portable Photosynthesis System (LI-COR, Lincoln, NE, USA) equipped with a red LED light source. The measurement protocol maintained standardized environmental conditions, including photosynthetic active radiation of 500 ± 50 μmol/m^2^·s, CO_2_ concentration of 400 μmol/mol, a gas flow rate of 500 μmol/s, leaf temperature of 25°C, and relative humidity of 55 ± 5%. All measurements were systematically performed between 9:00 and 11:00 a.m. under stable environmental parameters. Additionally, WUE was calculated using Equation 1.^23^

(1) $$\mathrm{WUE}=\frac{P_{n}}{\mathrm{Tr}}$$

### Chlorophyll and total nitrogen contents

In this experiment, a chlorophyll meter (TYS-4N, China) was used to measure the chlorophyll and total nitrogen contents in the rice leaves.^24^ (Put rice leaves at the detection point of the chlorophyll analyzer and read the screen data.)

### Selenium and cadmium bio transfer/bioconcentration factor

After digesting the roots, stems, and leaves of the rice samples, total Se and Cd contents were determined using the ICP-MS method.^25^^,^^26^ Additionally, the total Se and Cd transport coefficients, along with the concentration coefficient, were calculated using Equations 2 and 3.

2 $$\mathrm{TF}=\frac{\mathrm{leaf}\;(\mathrm{Se},\mathrm{Cd})}{\mathrm{Root}\;(\mathrm{Se},\mathrm{Cd})}$$

3 $$\mathrm{BCF}=\frac{\mathrm{leaf}\;(\mathrm{Se},\mathrm{Cd})}{\mathrm{Environ}\;(\mathrm{Se},\mathrm{Cd})}$$

### Dynamics electrophysiological sensors measurement and calculation

**Figure 1.**
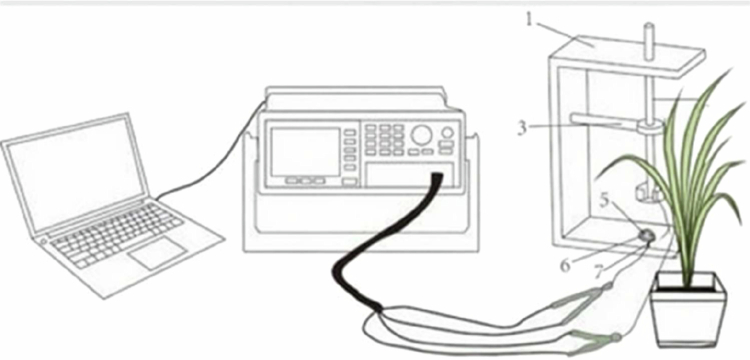
Schematic of the plant electrophysiological technology method: 1: holder, 2: cystosepiment, 3: plate electrode, 4: electrical conductor, 5: iron block, 6: plastic rod, and 7: bench hold.

Three weeks after rice treatment, rice plants exhibiting consistent growth and overall health under various treatments were selected for testing. The third fully expanded leaves of these plants were placed between parallel plates, with the measurement voltage set to 1.5 volts and the frequency adjusted to 3000 Hz to ensure accurate readings. The parallel capacitance sensors were configured in parallel mode for assessment using an LCR tester (LCR-6100, China, Figure 1).^27^ Under different clamping forces (F), the resistance (R), impedance (Z), and capacitance (CP) of the leaves were measured. For each clamping force, data were collected across 15–20 sets to maintain consistency. Ultimately, fifteen data sets were selected for further calculations to ensure reliable and statistically significant results in evaluating the electrical properties of the leaf tissues under the experimental conditions.

The Gibbs free energy (ΔG) is expressed by the equation: ΔG = ΔH +PV, where ΔH represents the internal energy of the plant leaf system, composed of cellular structures; P is the pressure applied to the plant cells; and V is the volume of these cells. In contrast, the energy stored in a capacitor is described by the equation: W = 1/2 U^2^ C, where W denotes the energy stored in the capacitor, U is the applied test voltage, and CP represents the physiological capacitance of the plant leaves. It can be established that this energy is equated to the work derived from the Gibbs free energy, leading to the relation W = ΔG. To quantify the pressure exerted on the plant cells, the equation P = F/S was used, where F is the clamping force and S is the effective area under the influence of the parallel plate capacitance sensor. Furthermore, the physiological capacitance C of the plant leaves is modeled to vary with changes in the clamping force F.^27^

(4) $$C=\frac{2∆H}{U^{2}}+\frac{2V}{{\mathrm{SU}}^{2}}F$$

In Equation 4, $y_{0}=\frac{2∆H}{U^{2}}$, $a=\frac{2V}{{\mathrm{SU}}^{2}}$ can be adjusted to reflect the variation in C of the plant leaves with respect to F, as described by Equation 2.

(5) $$C=y_{0}+aF$$

The capacitive reactance (Xc) was calculated using Equation 6:

(6) $$\mathrm{Xc}=\frac{1}{2\pi fC}$$

In Equation 6, π is 3.1416, and *f* is 3000 Hz.

Based on the values of R, Z, and Xc, the X_L_ of the plant leaves was analyzed using Equation 7.

(7) $$\frac{1}{{-X}_{L}}=\frac{1}{Z}-\frac{1}{R}-\frac{1}{\mathrm{Xc}}$$

Using the Gibbs free energy equation and the Nernst equation, the models relating R (Z/Xc/X_L_) to F were derived as shown in Equations 8–11.

(8) $$R=y_{1}+a_{1}e^{-b_{1}F}$$

(9) $$Z=y_{2}+a_{2}e^{{-b}_{2}F}$$

(10) $$\mathrm{Xc}=y_{3}+a_{3}e^{{-b}_{3}F}$$

(11) $$X_{L}=y_{4}+a_{4}e^{{-b}_{4}F}$$

When no clamping force (F = 0 N) is applied, the IR, IZ, IX_C_, and IX_L_ of the plant leaves can be calculated from Equations 12–15.

(12) $$\mathrm{IR}=y_{1}+a_{1}F$$

(13) $$\mathrm{IZ}=y_{2}+a_{2}F$$

(14) $$\mathrm{IXc}=y_{3}+a_{3}F$$

(15) $$IX_{L}=y_{4}+a_{4}F$$

The ICP of the plant leaves can be calculated using Equation 16.

(16) $$\mathrm{ICP}=\frac{1}{2\pi \;\mathrm{fIXc}}$$

In this experiment, plant electronic technology methods were employed to assess intracellular water and nitrogen metabolism in rice leaves, with equations referenced from previous studies.^27^^,^^28^ The IWHC of the plant leaves was calculated using Equation 17.

(17) $$\mathrm{IWHC}=\sqrt{{\left(\mathrm{ICP}\right)}^{3}}$$

The specific effective thickness (d) of the plant leaves was calculated using Equation 18.

(18) $$d=\frac{U^{2}a}{2}$$

The IWUE of the plant leaves was calculated using Equation 19.

(19) $$\mathrm{IWUE}=\frac{d}{\mathrm{IWHC}}$$

The IWHT of the plant leaves was calculated using Equation 20.

(20) IWHT=ICP×IZ

The WTR of the plant leaves was calculated using Equation 21.

(21) $$\mathrm{WTR}=\frac{\mathrm{IWHC}}{\mathrm{IWHT}}$$

The nutrient flux per unit area (UNF) of the plant leaves was calculated using Equation 22.

(22) $$\mathrm{UNF}=\frac{R}{\mathrm{IXc}}+\frac{R}{{\mathrm{IX}}_{L}}$$

Since nutrients are soluble in water, WTR and NTR are conceptually similar. Both rates are interconnected, as nutrient movement relies on water as the transport medium. Consequently, they are often assigned the same value, which is calculated using Equation 23. Furthermore, NTC represents the total efficiency of nutrient movement within the plant system under specific conditions. It is calculated as the product of UNF and NTR, as detailed in Equation 24.

(23) NTR=WTR

(24) NTC=UNF×NTR

The UAF can be calculated using Equation 25. Therefore, NAC is the product of UAF and NTR, as indicated in Equation 26.

(25) $$\mathrm{UAF}=\frac{\mathrm{IXc}}{IX_{L}}$$

(26) NAC=UAF×NTR

### Statistical analysis

The experimental data were evaluated for statistical differences using analysis of variance (ANOVA), and Tukey's test (*p* ≤ 0.05) was performed for pairwise comparisons between the different experimental treatments. The results are presented as the mean ± standard deviation (SD), and the figures were designed using Origin 2024 (64-bit).

## Results

### Growth parameters of rice

Table 2 presents the growth parameters of rice, including root, stem, leaf, and total biomass, with the following ranking: MSi (10 mM Si^4+^+8 μM Se^4+^+6 μM Cd^2+^) > LSi (3 mM Si^4+^+8 μM Se^4+^+6  μM Cd^2+^) > Sc (8 μM Se^4+^+6 μM Cd^2+^) > Ck (no Si, Se, Cd) > HSi (15 mM Si^4+^+8 μM Se^4+^+6 μM Cd^2+^) > Cd (6 μM Cd^2+^). The smallest root, stem, leaf, and total biomass were observed in the Cd treatment, while the largest values were recorded in MSi. Compared to Ck, MSi significantly increased the root, stem, leaf, and total biomass by 102.30%, 133.77%, 106.88%, and 120.54%, respectively.

**Table 2.** Biomass of roots, stems, and leaves in rice.

Group	Root (g)	Stem (g)	Leaves (g)	Total biomass (g)
Ck	2.61 ± 0.64c	7.15 ± 1.17cd	3.44 ± 0.34c	13.19 ± 1.69d
Cd	1.50 ± 0.13d	4.79 ± 0.14d	2.61 ± 0.09c	8.90 ± 0.30f
Sc	3.00 ± 0.47c	9.04 ± 0.39c	5.22 ± 0.34b	17.26 ± 0.77c
LSi	3.69 ±​ 0.32b	12.62​ ±​ 1.06b	6.31​ ±​ 0.45a	22.61​ ±​ 1.43b
MSi	5.27​ ±​ 0.26a	16.71​ ±​ 4.02a	7.12​ ±​ 1.37a	29.10​ ±​ 5.25a
HSi	1.91​ ±​ 0.33d	5.61​ ±​ 0.67d	3.14​ ±​ 0.29c	10.66​ ±​ 1.08de

Ck (no Si, Se, Cd); Cd (6​ μM Cd^2+^); Sc (8​ μM Se^4+^+6​ μM Cd^2+^); LSi (3​ mM Si^4+^+8​ μM Se^4+^+6​ μM Cd^2+^); MSi (10​ mM Si^4+^+8​ μM Se^4+^+6​ μM Cd^2+^); HSi (15​ mM Si^4+^+8​ μM Se^4+^+6​ μM Cd^2+^). Each value represents the mean ± standard deviation (*n* = 3), and different letters indicate significant differences by ANOVA and Tukey's test (*n* = 3, *p* ≤ 0.05) between different treatments.

### Chlorophyll and total nitrogen contents of rice leaves

Table 3 shows the chlorophyll and total nitrogen contents in rice leaves. The results indicated the following order for chlorophyll and total nitrogen contents: MSi > LSi > Sc > Ck > Cd > HSi. Both the chlorophyll and total nitrogen contents were highest in MSi and lowest in HSi. Compared to Ck, MSi resulted in increases of 11.97% in chlorophyll and 9.98% in total nitrogen, while HSi caused decreases of 9.25% in chlorophyll and 7.84% in total nitrogen.

**Table 3.** Chlorophyll and total nitrogen contents of rice leaves.

Group	Chlorophyll (mg/g)	Total nitrogen (mg/g)
Ck	44.78​ ±​ 1.01d	16.84​ ±​ 0.32d
Cd	41.36​ ±​ 0.47e	15.84​ ±​ 0.25e
Sc	48.36​ ±​ 0.30c	17.90​ ±​ 0.088c
LSi	49.32​ ±​ 0.33b	18.26​ ±​ 0.11b
MSi	50.14​ ±​ 0.23a	18.52​ ±​ 0.08a
HSi	40.64​ ±​ 0.59f	15.52​ ±​ 0.22f

Ck (no Si, Se, Cd); Cd (6​ μM Cd^2+^); Sc (8​ μM Se^4+^+6​ μM Cd^2+^); LSi (3​ mM Si^4+^+8​ μM Se^4+^+6​ μM Cd^2+^); MSi (10​ mM Si^4+^+8​ μM Se^4+^+6​ μM Cd^2+^); HSi (15​ mM Si^4+^+8​ μM Se^4+^+6​ μM Cd^2+^). Each value represents the mean ± standard deviation (*n* = 3), and different letters indicate significant differences by ANOVA and Tukey's test (*n* = 3, *p* ≤ 0.05) between different treatments.

### Selenium and cadmium transport and enrichment in different organs of rice

Total Se and Cd contents in various rice organs are presented in Tables 4 and 5. The total Se content followed this order: Sc > HSi > MSi > LSi > Cd > Ck, with the lowest total Se in Ck and the highest in Sc. Compared to Cd, Ck showed a decrease in total Se by 18.87%, while Sc significantly increased total Se by 34.34 times. The total Cd content followed the order: Cd > Sc > MSi > HSi > LSi > Ck, with the lowest total Cd in Ck and the highest in Cd. Compared to Cd, Ck exhibited a reduction in total Cd by 98.98%. Table 6 shows the bio-transfer factor of total Se (TF Se) in rice leaves, which followed this order: HSi > MSi > LSi > Sc > Ck > Cd. The TF Se was lowest in Cd and highest in HSi. Compared to Cd, the TF Se of HSi increased by 82.75%. The bio-transfer factor of total Cd (TF Cd) in rice leaves followed this order: Ck > HSi > LSi > Cd > MSi > Sc, with the smallest value in Sc and the largest in Ck. Compared to Cd, the TF Cd of Sc decreased by 29.32%, while the TF Cd of Ck increased by 8.13 times. The bio-concentration factor of total Se (BCF Se) was highest in HSi, followed by Sc, MSi, and LSi. The BCF Se was lowest in LSi and highest in HSi. The bio-concentration factor of total Cd (BCF Cd) followed this order: Cd > HSi > Sc > MSi > LSi, with the smallest BCF Cd in LSi and the largest in Cd. The BCF Cd of LSi decreased by 59.80% compared to Cd.

**Table 4.** Total selenium content in various organs of rice.

Group/total selenium content	Root (mg/kg)	Stem (mg/kg)	Leaves (mg/kg)
Ck	4.34 ± 0.17d	1.37 ± 0.06d	0.61 ± 0.04d
Cd	5.09 ± 0.03d	2.16 ± 0.11e	0.54 ± 0.03d
Sc	216.04 ± 5.92a	29.08 ± 0.69b	30.22 ± 0.97b
LSi	146.68 ± 6.04c	24.76 ± 0.88c	25.9 ± 0.69c
MSi	167.4 ± 4.79b	28.9 ± 0.57b	29.92 ± 0.97b
HSi	163.77 ± 4.84b	34.34 ± 1.29a	31.46 ± 0.53a

Ck (no Si, Se, Cd); Cd (6 μM Cd^2+^); Sc (8 μM Se^4+^+6 μM Cd^2+^); LSi (3 mM Si^4+^+8 μM Se^4+^+6 μM Cd^2+^); MSi (10 mM Si^4+^+8 μM Se^4+^+6 μM Cd^2+^); HSi (15 mM Si^4+^+8 μM Se^4+^+6 μM Cd^2+^). Each value represents the mean ± standard deviation (*n* = 3), and different letters in each value indicate significant differences by ANOVA and Tukey's test (*n* = 3, *p* ≤ 0.05) between different treatments.

**Table 5.** Total cadmium content in various organs of rice.

Group/total cadmium content	Root (mg/kg)	Stem (mg/kg)	Leaves (mg/kg)
Ck	4.24​ ±​ 0.12d	2.12​ ±​ 0.06f	0.51 ± .02e
Cd	618.5​ ±​ 14.41a	49.03​ ±​ 1.69a	8.22​ ±​ 0.19a
Sc	400.91​ ±​ 9.89b	20.42​ ±​ 0.56c	3.79​ ±​ 0.15c
LSi	200.91​ ±​ 4.9e	17.82​ ±​ 0.46d	3.31​ ±​ 0.1f
MSi	291.05​ ±​ 7.42c	19.09​ ±​ 1.15cd	3.80​ ±​ 0.11e
HSi	262.89​ ±​ 5.64d	22.51​ ±​ 0.6b	5.06​ ±​ 0.14b

Ck (no Si, Se, Cd); Cd (6​ μM Cd^2+^); Sc (8​ μM Se^4+^+6​ μM Cd^2+^); LSi (3​ mM Si^4+^+8​ μM Se^4+^+6​ μM Cd^2+^); MSi (10​ mM Si^4+^+8​ μM Se^4+^+6​ μM Cd^2+^); HSi (15​ mM Si^4+^+8​ μM Se^4+^+6​ μM Cd^2+^). Each value represents the mean ± standard deviation (*n* = 3), and different letters indicate significant differences by ANOVA and Tukey's test (*n* = 3, *p* ≤ 0.05) between different treatments.

**Table 6.** Total selenium and cadmium bio transfer/bioconcentration factor in rice leaves.

Group	TF Se (×10^2^)	TF Cd (×10^2^)	BCF Se	BCF Cd
Ck	13.72​ ±​ 0.02e	12.15​ ±​ 0.69a	NA	NA
Cd	10.32​ ±​ 0.22f	1.33​ ±​ 0.04cd	NA	12.19​ ±​ 0.28a
Sc	13.93​ ±​ 0.64d	0.94​ ±​ 0.01d	48.35​ ±​ 1.80ab	5.65​ ±​ 0.18c
LSi	17.64​ ±​ 0.39c	1.64​ ±​ 0.01bc	41.01​ ±​ 1.10c	4.90​ ±​ 0.15e
MSi	18.33​ ±​ 0.98b	1.30​ ±​ 0.01cd	47.37​ ​​​​​​​±​​​​​​​​ 1.54b	5.63​ ​​​​​​​±​​​​​​​​ 0.16d
HSi	18.86​ ​​​​​​​±​​​​​​​​ 0.67a	1.93​ ​​​​​​​±​​​​​​​​ 0.04b	49.81​ ​​​​​​​±​​​​​​​​ 0.84a	7.50​ ​​​​​​​±​​​​​​​​ 0.21b

Ck (no Si, Se, Cd); Cd (6 μM Cd^2+^); Sc (8 μM Se^4+^+6 μM Cd^2+^); LSi (3 mM Si^4+^+8 μM Se^4+^+6 μM Cd^2+^); MSi (10 mM Si^4+^+8 μM Se^4+^+6 μM Cd^2+^); HSi (15 mM Si^4+^+8 μM Se^4+^+6 μM Cd^2+^). Each value represents the mean ± standard deviation (*n* = 3), and different letters indicate significant differences by ANOVA and Tukey's test (*n* = 3, *p* ≤ 0.05) between different treatments.

### Photosynthesis

**Figure 2.**
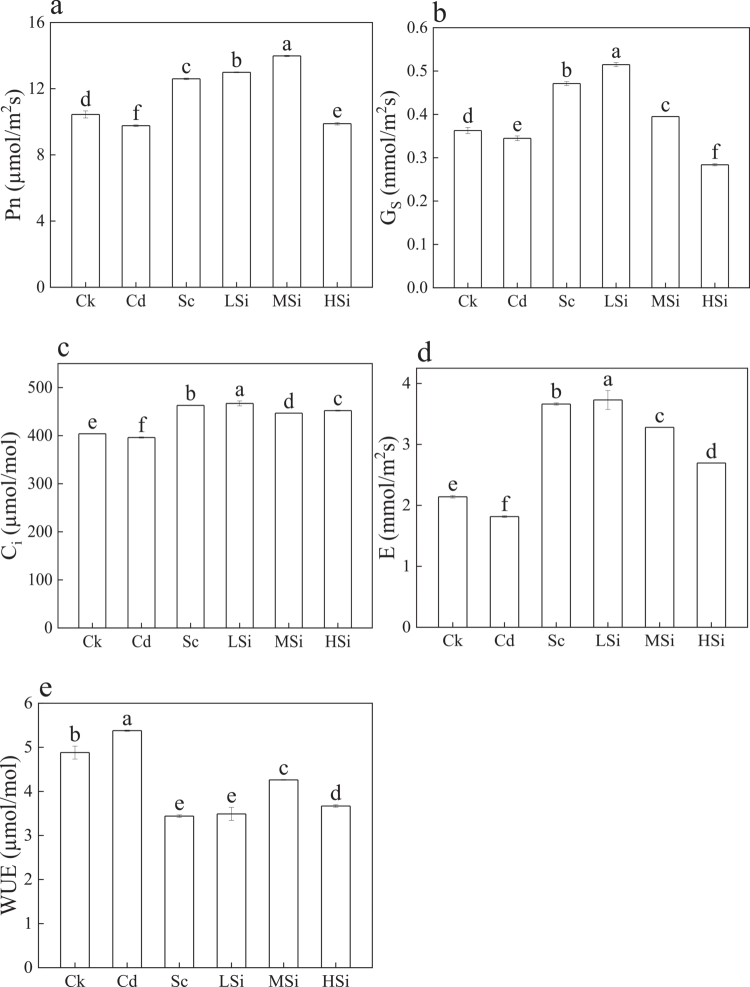
Photosynthesis parameters of rice leaves. a: net photosynthetic rate (Pn, µmol/m^2^s), b: stomatal conductance (G_S_, mmol/m^2^s), c: intercellular CO_2_ concentration (C_i_, µmol/mol), d: transpiration rate (E, mmol/m^2^s), and e: water use efficiency (WUE, µmol/mol). Ck (no Si, Se, Cd); Cd (6 μM Cd^2+^); Sc (8 μM Se^4+^+6 μM Cd^2+^); LSi (3 mM Si^4+^+8 μM Se^4+^+6 μM Cd^2+^); MSi (10  mM Si^4+^+8 μM Se^4+^+6 μM Cd^2+^); HSi (15 mM Si^4+^+8 μM Se^4+^+6 μM Cd^2+^). Each value represents the mean ± standard deviation (*n* = 3), and different letters indicate significant differences by ANOVA and Tukey's test (*n* = 3, *p* ≤ 0.05) between different treatments.

Figure 2 illustrates the photosynthesis parameters of rice leaves. Compared to Ck, the net photosynthesis rate (Pn) in MSi increased significantly by 33.86% (Figure 2a). G_S_ was highest in LSi, with a 41.90% increase, while HSi showed the lowest G_S_, with a 21.81% decrease (Figure 2b). C_i_ was lowest in Cd, with a 1.93% decrease, and highest in LSi, with a 15.64% increase (Figure 2c). The transpiration rate (Tr) was lowest in Cd, with a 15.17% decrease, and highest in LSi, with a 74.22% increase (Figure 2d). WUE was highest in Cd, with a 10.22% increase, and lowest in Sc, with a 29.52% decrease (Figure 2e).

### Electrophysiological sensors of rice leaves

**Figure 3.**
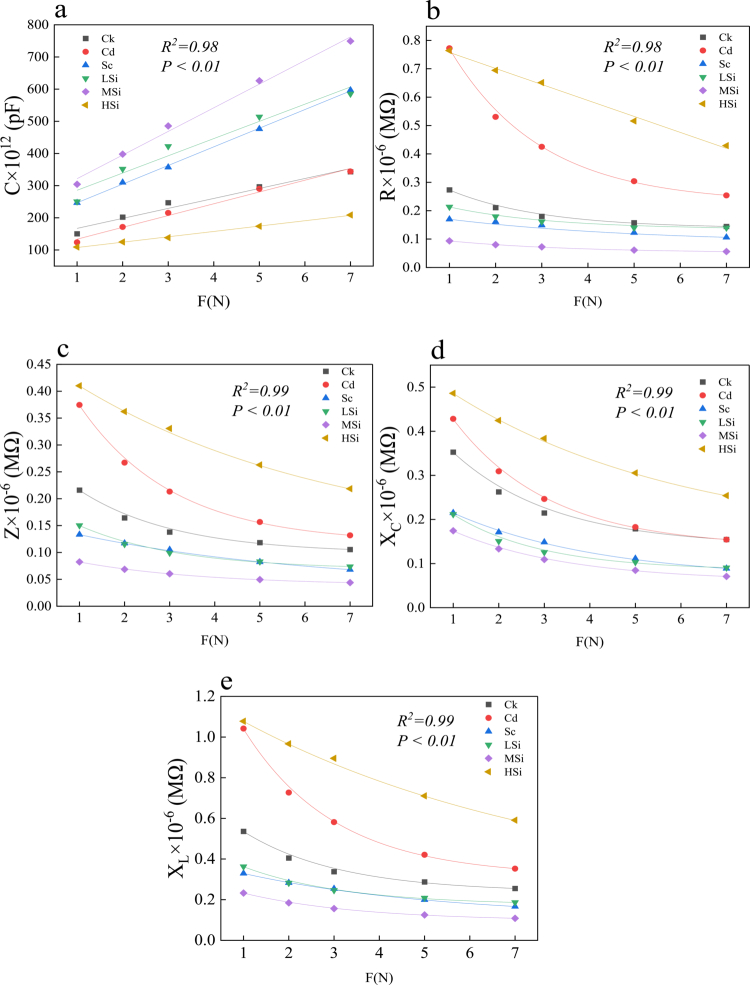
Parameter fitting of plant electrophysiological equations in rice leaves. F is the clamp force: 1 N, 2 N, 3 N, 5 N, and 7 N. a: C-capacitance, b: R-resistance, c: Z-impedance, d: Xc-capacitive reactance, and e: X_L_-inductive reactance. Ck (no Si, Se, Cd); Cd (6 μM Cd^2+^); Sc (8 μM Se^4+^+6 μM Cd^2+^); LSi (3 mM Si^4+^+8 μM Se^4+^+6 μM Cd^2+^); MSi (10 mM Si^4+^+8 μM Se^4+^+6 μM Cd^2+^); HSi (15 mM Si^4+^+8 μM Se^4+^+6 μM Cd^2+^). Each value represents the mean ± standard deviation (*n* = 3), and different letters in each value indicate significant differences by ANOVA and Tukey's test (*n* = 3, *p* ≤ 0.05) between different treatments.

This study presents the fitting curves of intrinsic capacitance (CP, Figure 3a), intrinsic resistance (R, Figure 3b), intrinsic impedance (Z, Figure 3c), intrinsic capacitive reactance (X_C_, Figure 3d), and intrinsic inductive reactance (X_L_, Figure 3e) of rice leaves in response to the clamping force (F). A significant correlation was observed (R^2^ > 0.99, p < 0.01) between CP, R, Z, X_C_, X_L_, and the applied clamping force on rice leaves.

### Intrinsic electrophysiological sensors of rice leaves

**Figure 4.**
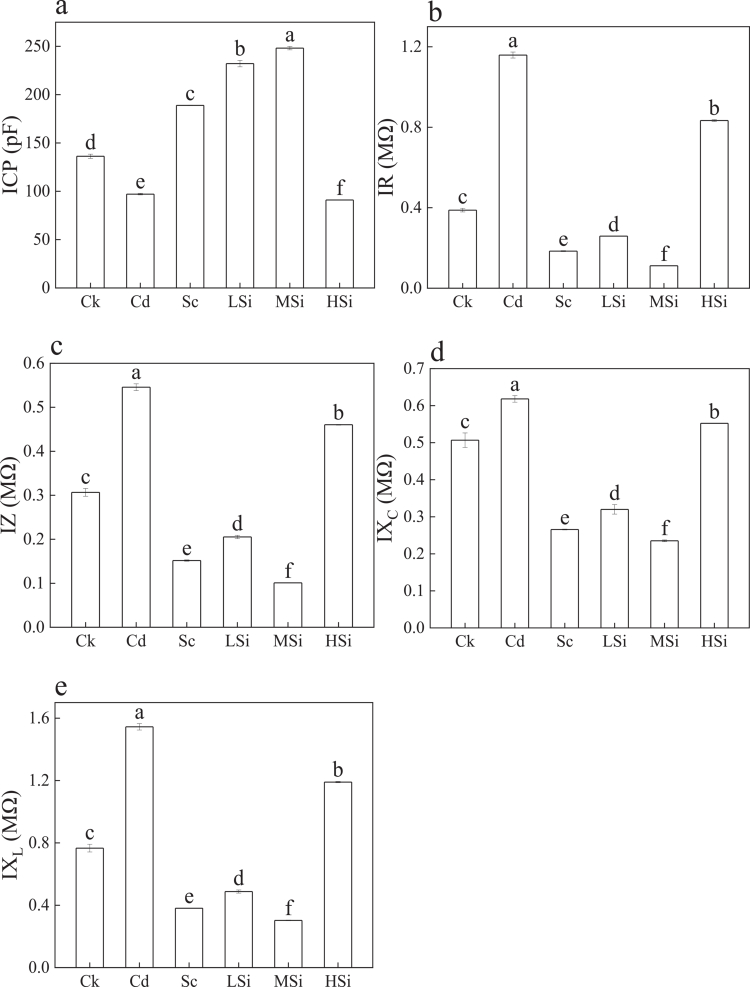
The plant electrophysiological sensors of rice leaves. a: ICP (intrinsic capacitance), b: IR (intrinsic resistance), c: IZ (intrinsic impedance), d: IX_C_ (intrinsic capacitive reactance), and e: IX_L_ (intrinsic inductive reactance). Ck (no Si, Se, Cd); Cd (6 μM Cd^2+^); Sc (8 μM Se^4+^+6 μM Cd^2+^); LSi (3 mM Si^4+^+8 μM Se^4+^+6 μM Cd^2+^); MSi (10 mM Si^4+^+8 μM Se^4+^+6 μM Cd^2+^); HSi (15 mM Si^4+^+8 μM Se^4+^+6 μM Cd^2+^). Each value represents the mean ± standard deviation (*n* = 3), and different letters in each value indicate significant differences by ANOVA and Tukey's test (*n* = 3, *p* ≤ 0.05) between different treatments.

Figure 4 illustrates the intrinsic electrophysiological sensors of rice leaves. In Figure 4a, the ICP followed the order: MSi > LSi > Sc > Ck > Cd > HSi. The highest ICP was observed in MSi, and the lowest in HSi. Compared to Ck, ICP increased by 82.11% in MSi and decreased by 33.21% in HSi. As presented in Figure 4b–e, IR, IZ, IX_C_, and IX_L_ were ranked as Cd > HSi > Ck > LSi > Sc > MSi. Compared to Ck, IR, IZ, IX_C_, and IX_L_ increased by 198.74%, 77.94%, 21.98%, and 101.53%, respectively, in Cd, whereas MSi showed decreases of 71.29%, 67.07%, 53.59%, and 60.48%, respectively.

### Intracellular water–nutrient metabolism based on electrophysiological sensors of rice leaves

**Figure 5.**
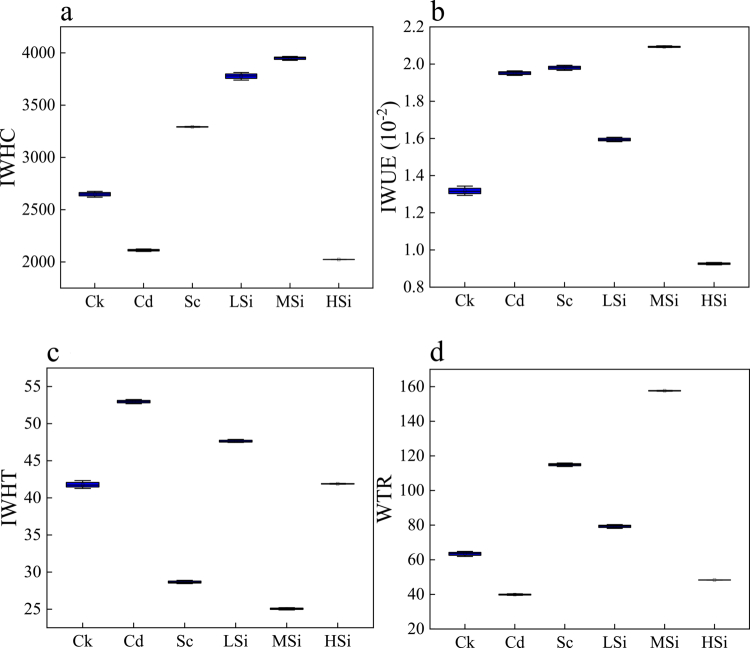
The dynamic intracellular water metabolism of rice leaves. a: IWHC (intercellular water-holding capacity), b: IWUE (intracellular water-use efficiency), c: IWHT (intracellular water-holding time), and d: WTR (water translocation rate). Ck (no Si, Se, Cd); Cd (6 μM Cd^2+^); Sc (8 μM Se^4+^+6 μM Cd^2+^); LSi (3 mM Si^4+^+8 μM Se^4+^+6 μM Cd^2+^); MSi (10 mM Si^4+^+8 μM Se^4+^+6 μM Cd^2+^); HSi (15 mM Si^4+^+8 μM Se^4+^+6 μM Cd^2+^). Each value represents the mean ± standard deviation (*n* = 3), and different letters in each value indicate significant differences by ANOVA and Tukey's test (*n* = 3, *p* ≤ 0.05) between different treatments.

**Figure 6.**
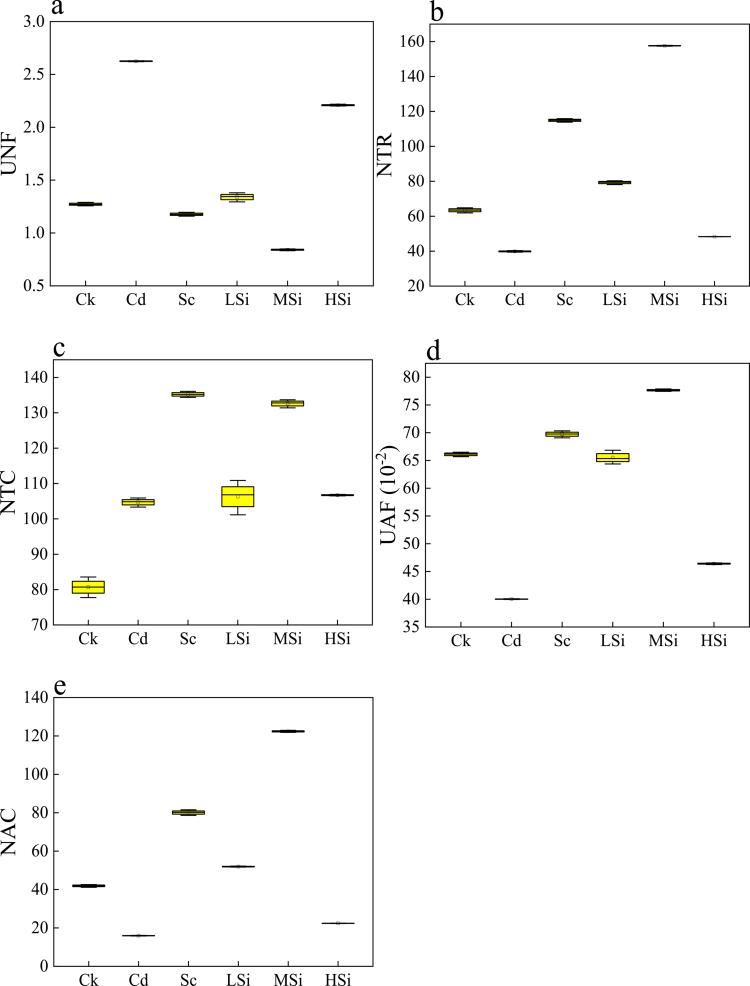
The dynamic intracellular nutrient metabolism of rice leaves. a: UNF (nutrient flux per unit area), b: NTR (nutrient translocation rate), c: NTC (nutrient translocation capacity), d: UAF (unit active transport flow of nutrients), and e: NAC (nutrient active translocation capacity). Ck (no Si, Se, Cd); Cd (6 μM Cd^2+^); Sc (8 μM Se^4+^+6 μM Cd^2+^); LSi (3 mM Si^4+^+8 μM Se^4+^+6 μM Cd^2+^); MSi (10 mM Si^4+^+8 μM Se^4+^+ 6 μM Cd^2+^); HSi (15 mM Si^4+^+8 μM Se^4+^+6 μM Cd^2+^). Each value represents the mean ± standard deviation (*n* = 3), and different letters in each value indicate significant differences by ANOVA and Tukey's test (*n* = 3, *p* ≤ 0.05) between different treatments.

Using the intrinsic electrical sensors of rice leaves (Figure 4), the intracellular water (Figure 5) and nutrient (Figure 6) metabolism of rice leaves were quantified. As presented in Figure 5, compared to Ck, MSi exhibited the highest levels of IWHC (Figure 5a), IWUE (Figure 5b), and WTR (Figure 5d), with increases of 49.13%, 58.35%, and 148.60%, respectively. In contrast, HSi showed the lowest levels of IWHC, IWUE, and WTR, with decreases of 23.81%, 29.55%, and 23.83%, respectively. Meanwhile, Cd exhibited the largest IWHT (Figure 5c), with an increase of 26.81%, while MSi displayed the smallest IWHT, with a decrease of 40%. As presented in Figure 6, compared to Ck, Cd had the highest UNF (Figure 6a), with an increase of 106.36%, while MSi exhibited the lowest UNF, with a decrease of 33.85%. MSi showed the highest NTR (Figure 6b), with an increase of 148.57%, while Cd had the lowest NTR, with a decrease of 37.10%. Sc exhibited the highest NTC (Figure 6c), with an increase of 67.60%, while MSi showed the highest UAF (Figure 6d), with an increase of 17.46%. Conversely, Cd had the lowest UAF, with a decrease of 39.46%. MSi displayed the highest NAC (Figure 6e), with an increase of 192.01%, while Cd showed the lowest NAC, with a decrease of 61.29%.

## Discussion

### Silicon–selenium interactively affects the growth of rice under cadmium stress

The Pn in rice increased under LSi and MSi treatments, while HSi resulted in a decrease compared to Cd. It has been reported that moderate levels of silicon can increase stomatal movement and photosynthesis, while high levels of silicon inhibit plant growth.^29^ Furthermore, rice biomass (Table 2) and photosynthesis (Figure 2) were highest in MSi, indicating that 8 μM Se^4+^ and 10 mM Si^4+^ significantly promoted rice growth. This effect is likely attributed to the increased formation of CdSe in the roots, which decreases Cd^2+^ transport from the roots to the shoots and leaves,^30^ thereby preventing a decline in photosynthesis.^31^ Additionally, the total Se content in rice leaves was higher in MSi (Table 4), which helped mitigate oxidative damage under high Cd stress. Se has also been shown to enhance the ascorbate‒glutathione (GSH‒AsA) cycle under Cd stress, preventing Cd translocation in plants.^32^ Furthermore, the chlorophyll and total nitrogen contents in rice leaves corresponded with biomass and photosynthesis,^33^^,^^34^ suggesting that MSi enhanced chlorophyll and total nitrogen contents in rice leaves.^35^ However, excessive silicon decreased stomatal movement in leaves,^36^ and inhibition of Pn at HSi was attributed to the high silicon concentration, which inhibited G_S_ and Tr, reducing stomatal opening and CO_2_ uptake, thereby limiting photosynthesis.^37^

### Silicon–selenium decreases cadmium accumulation in rice

In this study, compared to Cd, silicon-Se treatments promoted the total Se content (Table 4), TF Se, and BCF Se in rice plants (Table 6), while reducing the total Cd content (Table 5), TF Cd, and BCF Cd. The TF Cd of MSi was the lowest, indicating that 10 mM Si^4+^ and 8 μM Se^4+^ jointly inhibited Cd stress in rice plants. On one hand, Se promotes the formation of SeCd complexes, which inhibits Cd translocation from roots to shoots.^31^ On the other hand, silicon may enhance the formation of selenomethionine (SeMet), which decreases Cd translocation in plant leaves.^38^ Typically, while TF Se and BCF Se were highest at HSi (15 mM Si^4+^ and 8 μM Se^4+^), this treatment also exhibited high levels of TF Cd and BCF Cd. This result may be due to the formation of CdCO_3_–Si(CO_3_)_2_ rather than CdSe under excess silicon supply,^31^ leading to increased Cd translocation in rice leaves. Therefore, 10 mM Si^4+^ and 8 μM Se^4+^ effectively decreased Cd translocation in rice leaves in this experiment.

### High capacitance favors the cadmium resistance of rice leaves

Cell membrane potential changes in response to environmental variations, which manifests as alterations in electrophysiological sensors, resulting in differential ICP, IR, IZ, IX_C_, and IX_L_.^39^ In the present study, fitting equations for ICP, IR, IZ, IX_C_, IX_L_, and F demonstrated strong correlation (Figure 3), confirming the reliability of the electrophysiological equations.^40^ Therefore, when F equals 0, intrinsic electrophysiological sensor values, including ICP, IR, IZ, IX_C_, and IX_L_, were obtained for the rice leaves. The highest ICP and the lowest IR, IZ, IX_C_, and IX_L_ were observed at MSi, while the smallest ICP and the highest values for IR, IZ, IX_C_, and IX_L_ were found at Cd. This suggests that higher capacitance is positively correlated with rice growth.^40^ Larger ICP values are reported to indicate better water and nutrient metabolism, promoting plant growth.^41^^,^^42^ Furthermore, the decreased total Cd content was lowest at MSi (Table 5), contributing to the increased ICP in rice leaves.^43^

### Dynamic leaf intracellular water–nutrient metabolism using electrophysiological sensors of rice leaves

**Figure 7.**
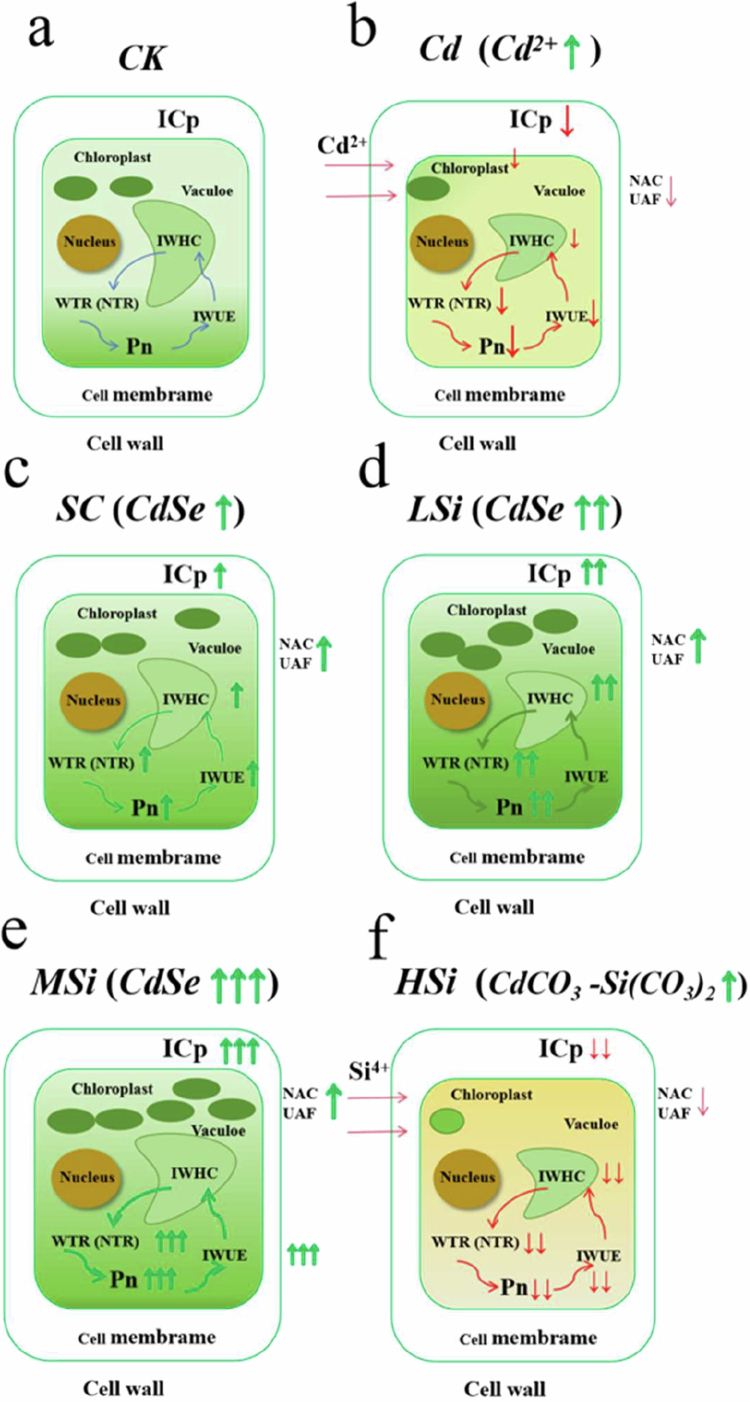
Joint interactions of silicon–selenium to intracellular water–nutrient metabolism of rice leaves under cadmium stress. ICP (intrinsic capacitance), IWHC (intracellular water holding capacity), IWUE (intracellular water use efficiency), WTR (water transfer rate), NTR (nutrient translocation rate), Pn (net photosynthetic rate), UAF (unit active transport flow of nutrient), NAC (nutrient active translocation capacity). Ck (no Si, Se, Cd); Cd (6 μM Cd^2+^); Sc (8 μM Se^4+^+6 μM Cd^2+^); LSi (3 mM Si^4+^+8 μM Se^4+^+6 μM Cd^2+^); MSi (10 mM Si^4+^+8 μM Se^4+^+6 μM Cd^2+^); HSi (15 mM Si^4+^+8 μM Se^4+^+6 μM Cd^2+^).

In this study, the highest IWHC, IWUE, and WTR were observed at MSi (8 μM Se^4+^ and 10 mM Si^4+^), while the lowest IWHT (Figure 5) was attributed to decreased Cd accumulation and increased rice plant growth. Increased biomass and photosynthesis were associated with enhanced intracellular water metabolism in plants.^44^ However, at HSi (8 μM Se^4+^ and 15 mM Si^4+^), leaf intracellular water metabolism was decreased, exhibiting the lowest IWHC, IWUE, and WTR due to inhibited rice growth and a decrease in Pn.^45^ Additionally, the IWHT at HSi significantly increased, indicating that mesophyll cells alleviated internal water shortage by extending water retention in rice leaves.^27^ Additionally, the intracellular nutrient metabolism in rice leaves was assessed using plant electrophysiological sensors. UNF represented the passive transport uptake capacity, while UAF, NTR, NTC, and NAC represented the active transport uptake capacity.^46^^,^^47^ The highest NTR, NTC, UAF, and NAC, along with the lowest UNF, were found at MSi (Figure 6). This was attributed to the significant promotion of growth and water utilization in rice leaves by MSi, suggesting that 10 mM Si^4+^ and 8 μM Se^4+^ facilitated the formation of binding proteins to increase the PC‒Cd complex and decrease the bioavailability of Cd. At high silicon levels, where vacuole volume is decreased, passive transport becomes the primary mode of nutrient translocation, which helps mitigate growth inhibition in plants.^48^ Thus, at HSi, the highest UNF and the lowest NTR and NAC were observed, indicating that 15 mM Si^4+^ and 8 μM Se^4+^ inhibited intracellular nutrient metabolism in rice leaves (Figure 7).

## Conclusion

This study, utilizing electrophysiological sensors, investigates the interaction between silicon and Se in dynamic intracellular water-nutrient metabolism in rice leaves under Cd stress. MSi promoted rice growth, whereas HSi inhibited it. MSi exhibited higher WTR, NAC, and NTR, enhancing Se translocation and SeCd formation, which in turn decreased Cd accumulation in rice leaves. In contrast, HSi suppressed growth and photosynthesis, likely due to decreased IWHC, NAC, and Se translocation, while potentially promoting the formation of CdCO_3_–Si(CO_3_)_2_ in rice leaves. Therefore, 10 mM Si^4+^ and 8 μM Se^4+^ increased intracellular water and nutrient metabolism in rice leaves to mitigate Cd stress, while 15  mM Si^4+^ and 8 μM Se^4+^ decreased rice growth by inhibiting leaf water and nutrient metabolism under Cd stress.

## Supplementary Material

Supplementary Material

## Abbreviations

CP: capacitance
R: resistance
Z: impedance
X_C_: capacitive reactance
X_L_: inductive reactance
ICP: intrinsic capacitance
IR: intrinsic resistance
IZ: intrinsic impedance
IX_C_: intrinsic capacitive reactance
IX_L_: intrinsic inductive reactance
IWHC: intracellular water holding capacity
IWHT: intracellular water holding time
IWUE: intracellular water use efficiency
WTR: water transfer rate
UNF: nutrient flux per unit area
UAF: active transport flow of nutrient
NTC: nutrient translocation capacity
NAC: nutrient active translocation capacity
NTR: nutrient translocation rate
TF: translocation factor
BCF: bioconcentration factor
