# Supplementary material for: Electrophysiological sensors reveal silicon–selenium interaction of dynamic leaf intracellular water–nutrient metabolism in rice under cadmium stress
Source: Plant Signal Behav. 2025 Aug 23;20(1):2547384. doi: 10.1080/15592324.2025.2547384 (PMC12377122; doi:10.1080/15592324.2025.2547384)
Supplement: Supplementary Material [file KPSB_A_2547384_SM2845.pdf]

Bullet Edits

EDITORIAL CERTIFICATE

This document certifies that the paper listed below was edited and proofread for proper English language, grammar, punctuation, spelling, and overall style by one or more than one highly qualified native speakers at Bullet Edits. All of the suggested amendments were tracked with the Microsoft Word "Track Changes" feature. Therefore, the author had the option to reject or accept each change individually.

Manuscript Title:

Electrophysiological sensors reveal silicon-selenium interaction of dynamic leaf intracellular water-nutrient metabolism in rice under cadmium stress

Anti-counterfeiting Code:

08ba39cf1e942365ceb22ff5f808ec1d

Date Issued:

2025-06-26 10:30:08

Bullet Edits is a registered company headquartered in the UK with a global presence. We offer a range of editing, proofreading services to authors. Our Ph.D. editors are all native English speakers from the USA and UK. Authors who work with Bullet Edits are guaranteed excellent language quality and timely delivery.

Address: BULLET EDITS LIMITED, 85 Great Portland Street, London,UK
Tel: UK. (+44)20457 70286 / US.(+1)312-313-9179
Web: www.bulletedits.cn/Email: info@bulletedits.cn
VAT: GB 378 9316 43

BULLET EDITS LIMITED ★ COMPANY NO. 13345261 SEAL ★ ENGLAND AND WALES
